# Patient perceptions regarding physician reimbursements, wait times, and out-of-pocket payments for anterior cruciate ligament reconstruction in Ontario

**DOI:** 10.1186/s40634-017-0076-6

**Published:** 2017-01-23

**Authors:** Muzammil Memon, Lydia Ginsberg, Darren de SA, Andrew Nashed, Nicole Simunovic, Mark Phillips, Matthew Denkers, Rick Ogilvie, Devin Peterson, Olufemi R. Ayeni

**Affiliations:** 1 Michael G. DeGroote School of Medicine, McMaster University, Hamilton, ON Canada; 2 Department of Science, McMaster University, Hamilton, ON Canada; 3 Division of Orthopaedic Surgery, Department of Surgery, McMaster University, Hamilton, ON Canada; 4 Department of Health Sciences, McMaster University, Hamilton, ON Canada; 5 Department of Clinical Epidemiology and Biostatistics, McMaster University, Hamilton, ON Canada; 6 McMaster University Medical Centre, 1200 Main St West, 4E15, Hamilton, ON L8N 3Z5 Canada

**Keywords:** Physician reimbursements, Anterior cruciate ligament, Patient perceptions, Wait times

## Abstract

**Background:**

Currently, there is a lack of knowledge regarding patient perceptions surrounding physician reimbursements, appropriate wait times, and out-of-pocket payment options for anterior cruciate ligament reconstruction (ACLR). Our objective was to determine the current state of these perceptions in an Ontario setting.

**Methods:**

A survey was developed and pretested to address patient perceptions about physician reimbursements, appropriate wait times, and out-of-pocket payment options for ACLR using a focus group of experts and by reviewing prior surveys. The survey was administered to patients in a waiting room setting.

**Results:**

Two hundred and fifty completed surveys were obtained (79.9% response rate). Participants responded that an appropriate physician reimbursement for ACLR was $1000.00 and that the Ontario Health Insurance Plan (OHIP) reimbursed physicians $700.00 for ACLR. Seventy-four percent of participants responded that the OHIP reimbursement of $615.20 for the procedure was either lower or much lower than what they considered to be an appropriate reimbursement for ACLR. Over 90% of participants responded that an ACLR should occur within 90 days of injury. Thirty-five percent of participants were willing to pay $750.00 out-of-pocket to have an ACLR done sooner, while 16.4% of participants were willing to pay $2500.00 out-of-pocket to travel outside of Canada for expedited surgery.

**Conclusion:**

This survey study demonstrates that patients’ estimates of both appropriate and actual physician reimbursements were greater than the current reimbursement for ACLR. Further, the majority of individuals report that the surgical fee for ACLR is lower than what they consider to be an appropriate amount of compensation for the procedure. Additionally, nearly all respondents believe that a ruptured ACL should be reconstructed within 90 days of injury. Consequently, a number of patients are willing to pay out-of-pocket for expedited surgery either in Canada or abroad. However, patients’ preferences for shorter wait times must be balanced with the known risk of arthrofibrosis associated with early ACLR.

**Electronic supplementary material:**

The online version of this article (doi:10.1186/s40634-017-0076-6) contains supplementary material, which is available to authorized users.

## Background

The anterior cruciate ligament (ACL) plays an important role in stabilizing the knee joint (American Academy Of Orthopaedic Surgeons 2009) However, when it is ruptured, it can result in a debilitating injury, especially for physically active individuals. If left unaddressed, an ACL injury can result in long-term consequences, including chronic knee instability, cartilage damage, and possibly osteoarthritis. An estimated 250,000 ACL injuries occur annually in Canada and the United states, which results in 100,000 ACL reconstructions (ACLR) being performed yearly (American Academy Of Orthopaedic Surgeons 2009; Hewison C). Thus, ACLR plays a major role in allowing for restoration of knee stability and healing after an injury.

Ontario’s health care system has many benefits, chiefly, its universal access allowing for all patients to have their basic health care needs met. However, this publicly funded, single payer system has often given rise to disagreements between provincial insurance plans (e.g. Ontario Health Insurance Plan, OHIP) and physician interest groups (e.g. Ontario Medical Association). Recently, in Ontario, the government has decreased funding for physician services by at least 6.95% (Canadian Medical Association 2016; Town of Caledon 2016; Ontario Ministry of Health and Long Term Care 2015). Similar decreases in funding towards Medicare spending have been observed for orthopaedic specialities in the United States and several studies have found that patients report that orthopaedic surgeons are reimbursed less than what they consider to be an appropriate remuneration (Tucker et al. 2013; Foran et al. 2012; Nagda et al. 2015). Currently, patient perceptions regarding physician reimbursements for ACLR is undocumented within an Ontario population.

Furthermore, due to limited resources, a challenge within the Ontario health care system is extensive wait times for elective surgery such as ACLR. The Ontario government has set a target for wait times to be under 182 days for arthroscopic knee surgery (Ministry of Health and Long Term Care 2016). However, on average, patients in Ontario will wait 210 days for an ACLR (Salci et al. 2015). In the climate of health care funding challenges and possible increased physician emigration, these wait times may worsen.

Thus, the primary objective of this study is to determine patient perceptions surrounding physician reimbursements for ACLR, including their perceptions surrounding the appropriateness of the current OHIP reimbursement for the procedure. The secondary objectives of this study are to determine patient perceptions surrounding appropriate wait times for the procedure and their willingness to pay out-of-pocket to have the procedure performed sooner.

## Methods

### Survey development

A focus group of Canadian orthopaedic surgeons who perform ACLR was formed to determine key parameters and indices to be included in the survey. These surgeons also reviewed prior surveys addressing patient perceptions of physician reimbursements for common orthopaedic procedures (Tucker et al. 2013; Foran et al. 2012; Nagda et al. 2015). Questions were designed to address patient perceptions surrounding physician reimbursements for ACLR, as well as patient perceptions surrounding appropriate wait times and out-of-pocket payment options.

### Survey pretesting and validity assessments

The survey was pretested within an independent group of five orthopaedic surgeons and assessed for appropriateness of content, ease of understanding, and comprehensiveness. As a result, the following sections were designed: (1) patient demographics and characteristics, (2) patient perceptions surrounding physician reimbursements for ACLR, (3) patient perceptions surrounding appropriate wait times, and (4) patients’ willingness to pay out-of-pocket to have the procedure performed sooner. Specifically, patient perceptions regarding the appropriateness of current OHIP reimbursement referenced the 2016 OHIP schedule of benefits which indicates that the fee for an ACLR is $615.20 (Ministry of Health and Long Term Care 2016). All authors were consulted once more for final review before the survey was finalized for administration to patients. Survey responses were collected using multiple choice options, as well as open-ended responses. See Additional file 1 for the complete survey.

### Survey administration

Between January and June 2016, 313 surveys were administered in the waiting rooms of three orthopaedic centers in the city of Hamilton, Ontario after receiving approval from the regional ethics review board (#20160669). The inclusion criteria for our study consisted of patients 18 years of age and older who were waiting for an appointment in the fracture clinic. Potential respondents were approached by data collectors and were asked to voluntarily complete an anonymous survey. Participants were oriented to the topic of the survey through an information sheet, which outlined the aims of the survey and provided pertinent background information regarding ACL anatomy, injury, and reconstruction (Appendix). If patients agreed to participate, they completed a consent form and were then given a blank survey. A data collector assisted patients if they had specific questions pertaining to the survey but did not direct their answers. Completed surveys were returned to the data collectors in a sealed envelope to maintain anonymity.

### Sample size calculation

The sample size required for our study was based on a calculation using Cochran’s sample size formula and Cochran’s correction formula for survey research studies, which can be combined to produce the following equation: $Sample\  Size=\frac{\frac{z^2\times p\left(1-p\right)}{e^2}}{1+\left(\frac{e^2\times p\left(1-p\right)}{e^2N}\right)},$ where population size = N; margin of error = e; z-score = z (Barlett et al. 2001). Our population size was 3507, based on the number of unique annual patients encountered in our clinics, the z-score was 1.65, the margin of error was 5%, and the estimate of variance (i.e. p(1-p)) was 0.25, which is standard for survey studies, as per Cochran’s sampling techniques (Barlett et al. 2001). After performing the calculation, the required sample size for this study was 250.

### Statistical analysis

For the purposes of statistical analysis, statistical significance was achieved when the *p*-value was less than 0.05. Descriptive statistics including medians, interquartile ranges (IQR), and proportions were used to summarize patient demographics and characteristics and patient perceptions regarding physician reimbursements, wait times, and out-of-pocket payments. Medians and IQR’s were used as measures of central tendency and variance, respectively, due to the large variation in patient responses to open-ended questions (e.g. patients’ estimates of OHIP reimbursements for ACLR). In instances where patient responses were discordant between successive questions (e.g. patient without a history of ACLR who stated that they were unhappy with the results of their ACLR), the initial patient response was used as the true patient characteristic (i.e. patient without a history of ACLR), and the discordant characteristic (i.e. unhappy with the results of ACLR) was adjusted to the most concordant response (i.e. no history of ACLR).

An independent samples *T*-test was used to compare mean responses regarding the amount that patients were willing to pay to have expedited surgery in Canada based on whether or not they were also willing to travel outside of Canada to have expedited surgery. Further, exploratory univariate analyses were conducted in order to test our hypotheses that the patient demographics and characteristics would be associated with the following six outcome variables regarding ACLR: (1) patient perceptions of appropriate physician reimbursements, (2) patient perceptions of current OHIP reimbursements, (3) patient perceptions surrounding the appropriateness of current OHIP reimbursement, (4) patient perceptions surrounding appropriate wait times, (5) patients’ willingness to pay out-of-pocket, and (6) patients’ willingness to travel outside of Canada and pay out-of-pocket. Any variable that showed significance in the univariate analysis was then used in a multivariable analysis to test the contribution of each of these variables to the six aforementioned outcome variables. Specifically, multiple linear regressions were used for the first two continuous outcome variables; ordinal logistic regressions were used for the third and fourth ordinal outcome variables; and multinomial logistic regressions were used for the fifth and sixth nominal outcome variables. Results were expressed using Beta coefficients, standard errors, 95% confidence intervals, p-values, and odds ratios.

## Results

### Patient demographics and characteristics

Three hundred and thirteen surveys were given to patients who met the inclusion criteria and 250 completed surveys were returned, which concluded the recruitment process and yielded a response rate of 79.9% fully completed surveys. The mean age (range) of participants was 38.8 years (18–92) and of the total respondents, 122 (48.8%) were male, 120 (48.0%) were female, and 8 (3.2%) did not specify their gender (Table 1). Further, 50 (20.0%) patients stated that they currently work or have previously worked in a healthcare setting. Additional demographic data from study participants is summarized in Table 1.

**Table 1:** Patient demographics and characteristics (*n* = 250)

Variable	N (%)
Mean Age	38.8 (range, 18-92)
Gender	
Male	122 (48.8%)
Female	120 (48.0%)
Prefer not to answer	8 (3.2%)
Education	
Did not graduate high school	18 (7.2%)
High school or high school equivalent	53 (21.2%)
Some college/university	91 (36.4%)
Undergraduate degree	43 (17.2%)
Graduate degree	35 (14.0%)
Prefer not to answer	10 (4.0%)
Income^a^	
< $40,922/year	42 (16.8%)
$40,923–$81,847/year	62 (24.8%)
$81,848–$150,000/year	69 (27.6%)
$150,001–$220,000/year	24 (9.6%)
> $220,000/year	11 (4.4%)
Prefer not to answer	42 (16.8%)
History of Employment in a Health Care Setting	
Yes	50 (20.0%)
No	197 (78.8%)
Prefer not to answer	3 (1.2%)
History of ACL or Severe Knee Injury	
Yes	120 (48.0%)
No	130 (52.0%)
History of (or awaiting) ACL Reconstruction	
Yes (history of ACL reconstruction)	53 (21.2%)
Yes (awaiting ACL reconstruction)	11 (4.4%)
No	186 (74.4%)
Wait Time Experienced for ACL Reconstruction	
Less than 5 days	4 (7.5%^b^)
5–21 days	0 (0.0%^b^)
21–90 days	11 (20.8%^b^)
90–180 days	21 (39.6%^b^)
Greater than 180 days	17 (32.1%^b^)
Not applicable	197 (78.8%^c^)
Time Passed Since ACL Reconstruction	
Less than 7 days	11 (20.8%^b^)
7 – 28 days	8 (15.1%^b^)
28 – 180 days	17 (32.1%^b^)
180 – 365 days	4 (7.5%^b^)
Greater than 365 days	13 (24.5%^b^)
Not applicable	197 (78.8%^c^)
Happy with Results of ACL Reconstruction	
Yes	33 (62.3%^b^)
No	5 (9.4%^b^)
Unsure	15 (28.3%^b^)
Not applicable	197 (78.8%^c^)
Satisfied with Current Orthopaedic Experience	
Very satisfied	75 (30.0%)
Satisfied	71 (28.4%)
Neutral	32 (12.8%)
Dissatisfied	4 (1.6%)
Very dissatisfied	0 (0.0%)
Prefer not to answer or have not had an orthopaedic experience	68 (27.2%)

^a^Income stratified based on Ontario provincial income tax brackets (Canada Revenue Agency 2016)

^b^Percentage based on individuals who have had an ACL reconstruction

^c^Percentage based on total number of participants

**Fig. 1:**
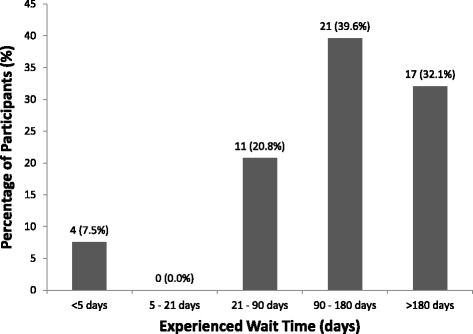
Wait time experienced by patients, N (%)

Of the total respondents, 120 (48.0%) stated that they currently have or have had an ACL injury or severe knee injury, 53 (21.2%) stated that they have had an ACLR in the past, and 11 (4.4%) stated that they are waiting for an ACLR (Table 1). With regards to the wait times experienced by participants who have had an ACLR, 4 (7.5%) waited less than 5 days, 0 (0.0%) waited between 5 and 21 days, 11 (20.8%) waited between 21 and 90 days, 21 (39.6%) waited between 90 and 180 days, and 17 (32.1%) waited greater than 180 days (Fig. 1). Further, 33 (62.3%) patients who have had an ACLR in the past stated that they were happy with the results, 5 (9.4%) said they were unhappy, and 15 (28.3%) said they were unsure. In general, 146 (58.4%) participants stated that they were either satisfied or very satisfied with their current orthopaedic experience, 32 (12.8%) stated that they were neutral, 4 (1.6%) were either dissatisfied or very dissatisfied, and 68 (27.2%) preferred not to answer or have not had an orthopaedic experience.

### Physician reimbursements

When patients were asked what they thought was an appropriate reimbursement for orthopaedic surgeons to receive for an ACLR, the median (IQR) value reported was $1000.00 (1800.00) (Table 2). A multivariable linear regression demonstrated that the presence of the following patient demographics and characteristics correlated significantly with larger patient estimates of appropriate physician reimbursements for ACLR: participants who did not graduate from high school (coefficient = 2500, *p* = 0.002); participants who had an income of less than $40,922 a year (coefficient = 1711, *p* = 0.001); participants who have previously been or currently are employed in a health care setting (coefficient = 2064, *p* < 0.000); participants who were happy (coefficient = 1524, *p* = 0.004) or unsure (coefficient = 1673, *p* = 0.027) regarding the results of their ACLR, respectively; and participants who were very satisfied with their orthopaedic experience (coefficient = 2295, *p* < 0.000).

**Table 2:** Physician reimbursements, out-of-pocket payments, and wait times

Variable	N (%)	Median (IQR)
What do you think is a reasonable fee that an orthopaedic surgeon should receive to perform an ACL reconstruction surgery?	250 (100%)	$1000.00 (1800)
How much do you estimate that OHIP actually pays an orthopaedic surgeon to perform an ACL reconstruction surgery?	250 (100%)	$700.00 (500)
What do you think is an appropriate wait time for an ACL reconstruction surgery after a diagnosis of an ACL injury?		
As soon as possible (including being placed on the emergency/after-hours list)	94 (37.6%)	
Within 21 days	62 (24.8%)	
Within 90 days	70 (28.0%)	
Within 180 days	18 (7.2%)	
Within 365 days	5 (3.3%)	
Never	1 (0.4%)	
Would you be willing to pay out-of-pocket to have an ACL reconstruction surgery sooner?		
Yes	87 (34.8%)	$750.00 (1500.00)
No	107 (42.8%)	
Unsure	56 (22.4%)	
Would you be willing to travel outside of Canada and pay out-of-pocket to have ACL reconstruction surgery sooner than when you can have it here?		
Yes	41 (16.4%)	$2500.00 (3750.00)
No	151 (60.4%)	
Unsure	58 (23.2%)	

Further, patients estimated that OHIP reimburses physicians a median (IQR) amount of $700.00 (500.00) for the procedure. A multivariable linear regression demonstrated that the presence of the following patient demographics and characteristics correlated significantly with larger patient estimates of OHIP reimbursements for ACLR: participants who did not graduate from high school (coefficient = 1624, *p* = 0.019); participants who had an income of less than $40,922 a year (coefficient = 1638, *p* < 0.000); participants who have previously been or currently are employed in a health care setting (coefficient = 1675, *p* < 0.000); participants who were happy with the results of their ACLR (coefficient = 1461, *p* = 0.001); and participants who were very satisfied with their orthopaedic experience (coefficient = 1489, *p* < 0.000).

**Fig. 2:**
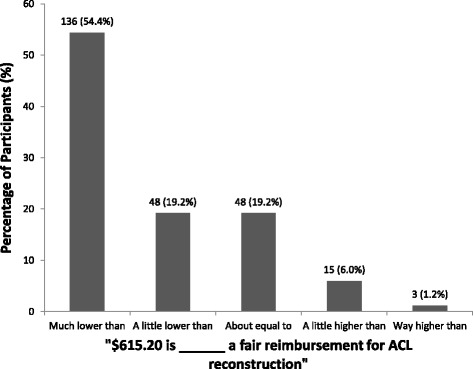
Patient perceptions surrounding the appropriateness of the current OHIP reimbursement for ACL reconstruction, N (%)

When the actual OHIP reimbursement was revealed to patients, 136 (54.4%) participants stated that it was much lower than what they considered to be an appropriate reimbursement for an ACLR, 48 (19.2%) stated that it was a little lower, 48 (19.2%) stated that it was about equal to an appropriate reimbursement for the procedure, 15 (6.0%) stated that it was a little higher, and 3 (1.2%) participants stated that it was way higher than an appropriate reimbursement for an ACLR (Fig. 2). An ordinal logistic regression demonstrated that an increase in, or presence of, the following patient demographics and characteristics correlated significantly with the opinion that physicians were reimbursed more than adequately: participants’ age (odds ratio = 1.05, *p* < 0.000) and participants who have had a previous ACL or severe knee injury (odds ratio = 1.99, *p* = 0.021). Whereas, the presence of the following patient demographics and characteristics correlated significantly with the opinion that physicians were reimbursed inadequately: participants who had a neutral orthopaedic experience (odds ratio = 0.21, *p* < 0.000) and participants who preferred not to comment on their orthopaedic experience (odds ratio = 0.37, *p* = 0.008).

### Wait times

**Fig. 3:**
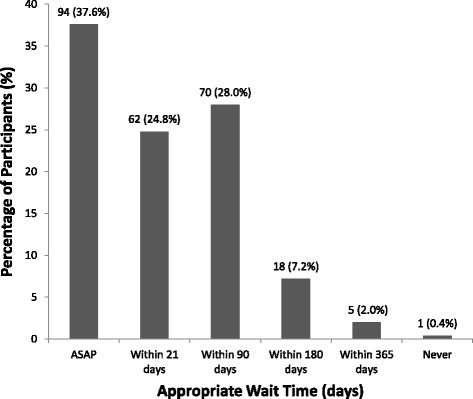
Patient perceptions regarding appropriate wait times for ACL reconstruction, N (%)

With regards to wait times, 94 (37.6%) patients stated that a torn ACL should be surgically reconstructed as soon as possible (including being placed on the emergency/after-hours list), whereas 62 (24.8%) stated that an appropriate wait time was 21 days or less, 70 (28.0%) said 90 days or less, 18 (7.2%) said 180 days or less, 5 (2.0%) said 365 days or less, and only 1 (0.4%) patient stated that an ACL should never be surgically reconstructed (Fig. 3). An ordinal logistic regression resulted in a statistically significant correlation between longer wait times and participants’ ages (odds ratio = 1.02, *p* = 0.005), which demonstrated that as participants’ ages decreased, they were more likely to prefer shorter wait times, and as participants’ ages increased, they were less opposed to longer wait times.

### Out-of-pocket

When patients were asked if they would be willing to pay out-of-pocket to have an ACLR, 87 (34.8%) said yes, 107 (42.8%) said no, and 56 (22.4%) said that they were unsure (Table 2). Of the patients who stated that they would pay out-of-pocket for the procedure, the median (IQR) amount they were willing to pay was $750.00 (1500.00). Moreover, when patients were asked if they would be willing to travel outside of Canada and pay out-of-pocket to have an ACLR, 41 (16.4%) said yes, 151 (60.4%) said no, and 58 (23.2%) said that they were unsure. If patients stated they were willing to pay to travel outside of Canada to have the procedure performed, the median (IQR) amount they were willing to pay for this option was $2500.00 (3750.00). Further, patients who were willing to travel outside of Canada for an expedited ACLR were willing to pay a median (IQR) amount of $2000.00 (2000.00) out-of-pocket to have the procedure done in Canada, whereas patients who were unwilling to travel outside of Canada were willing to pay $600.00 (625.00) out-of-pocket to have the procedure done in Canada, and this difference was statistically significant (*p* < 0.000). A multinomial logistic regression did not demonstrate any significant correlations between patient demographic or characteristic variables and patients’ willingness to pay out-of-pocket for an expedited ACLR both within and outside of Canada.

## Discussion

### Key findings

The main finding of our study was that the majority of patients (73.6%) responded that the amount that physicians were reimbursed by OHIP for an ACLR (i.e. $615.20) was either lower or much lower than what they considered to be an appropriate reimbursement for the procedure (i.e. $1000.00). Similar results were obtained by Tucker et al., who found that 62% of patients reported that surgeons should be reimbursed greater than the Medicare amount for total hip or knee arthroplasty (Tucker et al. 2013). Furthermore, patients in our study estimated that OHIP reimbursed physicians $700.00 for the procedure, which was only 13.8% greater than the actual OHIP reimbursement. This finding is inconsistent with several studies, including one by Nagda et al., which found that patients overestimated Medicare reimbursements for total shoulder arthroplasty and rotator cuff repair by 339 and 299%, respectively (Tucker et al. 2013; Foran et al. 2012; Nagda et al. 2015). Therefore, our findings, compared to other similar studies, demonstrate that patients in our study have a reasonable understanding of physician remuneration.

Our study also indicated a trend in patient preference towards shorter wait times for ACLR, with 226 (90.4%) patients reporting that an ACLR should occur within 90 days of injury. This is not surprising as a recent study by Salci et al. found that patients who experienced longer wait times were more likely to feel a sense of deterioration in their physical health and lose their jobs or be placed on modified duties (Salci et al. 2015). Further, a systematic review by Saltzman et al., which compared the costs associated with early versus delayed ACLR found that delayed ACLR was $1574 more expensive than an early procedure (Saltzman et al. 2015). Moreover, several studies have demonstrated that early ACLR yields better outcomes over delayed reconstruction, including a higher rate of medial meniscal repair, decreased loss of muscle function, and decreased risk of subsequent cartilage lesions and meniscal tears (Krutsch et al. 2015; Eriksson & Barenius B 2016; Granan et al. 2009). Our findings also demonstrated that patients’ actual experiences with wait times for ACLR were different compared to their expectations. Figures 1 and 3 illustrate the mismatch between patients’ expectations for efficiency and the current wait times in our health care system, as the trends are near mirror images.

Although patients prefer minimizing the time delay from injury to surgery, DeHaven et al. describe that early ACLR predisposes patients to an increased risk of arthrofibrosis with an incidence ranging from 4 to 35% (DeHaven et al. 2002). Further, Meighan et al. randomized 31 patients to early (i.e. within 2 weeks) versus delayed (i.e. between 8 and 12 weeks) ACLR and found that the range of flexion was reduced for the early group throughout the 52-weeks of follow-up. Moreover, they found that the mean quadriceps power and total work were reduced in the early group, with significant difference observed between the two groups at the 12-week follow-up (Meighan et al. 2003). These risks associated with early ACLR must be weighed against patients’ desire for shorter wait times when considering optimal timing of surgery.

Finally, 87 (34.8%) patients surveyed were willing to pay $750.00 out-of-pocket to have an ACLR performed sooner. Further, 41 (16.4%) respondents were willing to pay $2500.00 out-of-pocket for expedited surgery abroad, and this same group of patients was willing to pay $2000.00 for expedited surgery within Canada. These findings are in contrast to a Canadian study by O’Hara et al., which found that patients were strongly averse to paying out-of-pocket in order to reduce wait times for total shoulder arthroplasty (O’Hara et al. 2016). However, the reason for this difference was likely because their study surveyed an older population, living with a chronic arthritic condition, who had become adept at performing their daily activities with their unaffected arm (O’Hara et al. 2016). A similar finding was identified in our study as older patients were less opposed to longer wait times and younger patients were more likely to prefer shorter wait, which may possibly be attributed to decreasing activity requirements with age. Further, a 2014 survey demonstrated that 52,000 Canadians, including 2520 orthopaedic patients, travelled outside of Canada to receive health care services sooner despite the large out-of-pocket expense (e.g. $26,805 for total knee replacements) (Barua & Ren F 2015; Bell et al. 1998). Collectively, these findings demonstrate that there is a desire amongst some patients for shorter wait times and patients are increasingly willing to pay out-of-pocket in order to receive timely health care services, including elective surgery.

### Limitations

A potential source of bias was that patients completed the survey prior to their appointments with their orthopaedic surgeon, and although they were told that the results would be anonymous, the setting of the survey may have impacted patients’ responses. Also, our survey was administered in paper format where the true OHIP reimbursement was listed on the final page to avoid influencing prior responses. Although patients were asked not to proceed to the final question until completing all prior questions, it is difficult to ascertain compliance. Further, as in any cross-sectional study design, our understanding of patient perceptions are limited to only the time period of our data collection, whereas patient perceptions are dynamic and any changes cannot be accounted for in our study. Additionally, although we had a high response rate of 79.9%, it is unclear how the 20.1% of surveys that were incomplete would have influenced the results. Finally, there were some limitations due to certain aspects of our study sample as it was gathered from only academic hospitals in one city, and participants reported a greater household income compared to the income distributions information collected by government sources, which may limit the generalizability of our results (Bell et al. 1998).

### Strengths

There are several strengths to our study, including certain aspects of our study sample, as it was recruited from multiple hospitals and was very diverse with regards to: level of education and percentage of participants previously employed in a healthcare setting, both of which corresponded closely with frequency distributions from government statistics sources (Statistics Canada 2016; Statistics Canada 2015). Further, our study sample was diverse with regards to patients with a history of ACL injury and reconstruction, which allowed for comprehensive multivariable regression analyses between several important variables. Moreover, our primary outcome variables inquiring about patient perceptions of costs associated with ACLR allowed participants to provide open-ended responses and thus allowing for more comprehensive data collection. Finally, our study design is robust as it is one of the few to have incorporated a sample size calculation a priori and pretested all questions with a panel of content experts.

### Future directions

There is a need for more studies investigating patient perceptions regarding physician reimbursements, wait times, and out-of-pocket payments for orthopaedic procedures in Canadian settings, as this is one of the few studies of its kind. Future studies can answer questions that arose in our study, including the reasons why patients were unsure regarding their own satisfaction with the results of their ACLR, as well as the reasons why younger patients were more likely to prefer shorter wait times while older patients were less opposed to longer wait times. Moreover, further research should be conducted to assess the study’s outcomes in a more general patient population, including non-orthopaedic patients from a variety of medical specialties. Also, future studies must be done in more varied settings, and data should be collected in a longitudinal manner to assess how patient perceptions evolve over time.

## Conclusion

This survey study demonstrates that patients’ estimates of both appropriate and actual physician reimbursements were greater than the current reimbursement for ACLR. Further the majority of individuals report that the surgical fee for ACLR is lower than what they consider to be an appropriate amount of compensation for the procedure. Additionally, nearly all respondents believe that a ruptured ACL should be reconstructed within 90 days of injury, which is significantly lower than the government’s benchmark of 182 days. Consequently, a number of patients are willing to pay out-of-pocket for expedited surgery either in Canada or abroad. These results inform surgeons of patient perceptions regarding anterior cruciate ligament reconstruction, including physician reimbursements, wait times, and out-of-pocket payments. Future studies should be performed to see how these perceptions differ by setting and how they evolve over time.

## Appendix

### Background on Anterior Cruciate Ligament Reconstruction

#### Background

An orthopaedic surgeon is a highly trained surgeon who operates on muscles and the skeleton, which includes the tissue (ligaments) that connects bones to other bones. It takes 13 to 15 years of education and training after high school to become an orthopaedic surgeon.

The anterior cruciate ligament, otherwise known as the ACL, is a piece of tissue in the knee joint. The ACL keeps the knee stable. The ACL limits twisting motions on the knee and prevents the shinbone from sliding out in front of the thighbone.

The ACL is one of the most common ligaments injured in the knee. One form of treatment for an injured ACL is surgery. During surgery, an orthopaedic surgeon reconstructs the ACL. The surgery to reconstruct an ACL takes about 2 h.

#### OHIP Reimbursement

One type of surgery that OHIP reimburses surgeons for includes total knee replacements. This surgery takes about 2 h. A surgeon receives $838.00 for performing a total knee replacement.

OHIP also reimburses surgeons for performing surgery on broken ankles to put the broken pieces of bone together and hold them with hardware like pins, screws, and plates. This type of surgery takes about 2 h. Surgeons receive $237.50 for these operations.

In addition to the cost of the surgeon’s services, other related costs for surgeries may include:

- the nurses’ services before, during, and after the operation
- the anaesthesiologist’s services
- medications used during the operation
- equipment/materials used during the operation
- operating room costs

## Additional file

Additional file 1: Survey – Patient Perceptions Surrounding Anterior 441 Cruciate Ligament Reconstruction. (DOCX 13 kb)

## Abbreviations

ACL: Anterior cruciate ligament
ACLR: Anterior cruciate ligament reconstruction
IQR: Interquartile range
OHIP: Ontario health insurance plan
