# Supplementary material for: Patient perceptions regarding physician reimbursements, wait times, and out-of-pocket payments for anterior cruciate ligament reconstruction in Ontario
Source: J Exp Orthop. 2017 Jan 23;4:1. doi: 10.1186/s40634-017-0076-6 (PMC5256624; doi:10.1186/s40634-017-0076-6)
Supplement: Additional file 1: — Survey – Patient Perceptions Surrounding Anterior 441 Cruciate Ligament Reconstruction. (DOCX 13 kb) [file 40634_2017_76_MOESM1_ESM.docx]

*Additional file 1: Survey – Patient Perceptions Surrounding Anterior Cruciate Ligament Reconstruction*

Demographics

1.     Your age: _________________

2.     Your gender (circle one):   Male             Female          I prefer not to answer

3.     What is your highest level of education? (circle one)

a.     Did not graduate from High School

b.     High School or High School Equivalent

c.     Some College/University

d.     Undergraduate Degree

e.     Graduate Degree

f.      I prefer not to answer

4.     What was your approximate household income last year? (circle one)

a)     Less than $40,922 a year

b)    $40,923 — $81,847 a year

c) $81,848 — $150,000 a year

d) $150,001 — $220,000 a year

e) Greater than $220,000 a year

f) I prefer not to answer

5.     Have you worked or do you currently work in a healthcare setting? (circle one)

a.     Yes

b.     No

c.     I prefer not to answer

Your ACL Experience

Diagnosis

8.     Do you currently have or have you ever had an ACL injury or severe knee injury? (circle one)

a.     ACL injury

b.     Severe knee injury

Surgery

9.     Are you currently waiting for an ACL reconstruction surgery or have you ever had ACL reconstruction surgery? (circle one)

a.     Yes

b.     No

10.  How long did you have to wait for your ACL reconstruction surgery after you were told that you had an ACL injury?

a.     Less than 5 days

b.     5 — 21 days

c.     21 days — 3 months

d.     3 — 6 months

e.     Greater than 6 months

f.      I have never had an ACL injury and/or an ACL reconstruction surgery.

11.  How much time has passed since your ACL reconstruction surgery?

a.     Less than 1 week

b.     1—4 weeks

c.     1—6 months

d.     6—12 months

e.     Greater than 1 year

f.      I have never had an ACL reconstruction surgery.

Results

12.  If you have had an ACL reconstruction surgery in the past, are you happy with the results? (circle one)

a.     Yes

b.     No

c.     Unsure

d. I have never had an ACL reconstruction surgery.

13.  How satisfied are you with your current orthopaedic experience? (circle one)

a.     Very satisfied

b.     Satisfied

c.     Neutral

d.     Dissatisfied

e.     Very dissatisfied

f.      I Prefer Not To Answer or Have Not Had an Orthopaedic Experience

Reimbursement for Anterior Cruciate Ligament (ACL) Reconstruction

14.  What do you think is a reasonable fee that an orthopaedic surgeon should receive to perform an ACL reconstruction surgery? This excludes other costs related with the surgery.

$ _______________________ (please write the amount here)

15.  How much do you estimate that OHIP actually pays an orthopaedic surgeon to perform an ACL reconstruction surgery? This excludes other costs related with the surgery.

$ _______________________ (please write the amount here)

16.  Would you be willing to pay out-of-pocket to have an ACL reconstruction surgery sooner? This payment includes the cost of the surgeon’s services, the nurses’ services, the anaesthesiologist’s services, equipment/materials, and operating room time.

a.     Yes, I would pay up to $ _______________________ (please write the amount here)

b.     No

c.     Unsure

17.  Would you be willing to travel outside of Canada and pay out-of-pocket to have ACL reconstruction surgery sooner than when you can have it here? This payment includes the cost of the surgeon’s services, the nurses’ services, the anaesthesiologist’s services, equipment/materials, and operating room time, and excludes the cost of travel.

a.     Yes, I would pay up to $ _______________________ (please write the amount here)

b.     No

c.     Unsure

18.  Given that the risk of injury severity increases when surgery for an ACL injury is SIGNIFICANTLY delayed, what do you think is an appropriate wait time for an ACL reconstruction surgery after a diagnosis of an ACL injury?

a.     As soon as possible (including being placed on the emergency/after-hours list)

b.     Within 21 days

c.     Within 3 months

d.     Within 6 months

e.     Within a year

f.      Never

*Please answer questions 1-18 before going to the next page.*

Actual OHIP Reimbursement

19.  On average, the Ontario Health Insurance Plan (OHIP) pays an orthopaedic surgeon $615.20 for performing an ACL reconstruction surgery.  This excludes other costs related with the surgery. (Circle one)

a.     $615.20 is much lower than what a surgeon should make

b.     $615.20 is a little lower than what a surgeon should make

c.     $615.20 is about what a surgeon should make

d.     $615.20 is a little higher than what a surgeon should make

e.     $615.20 is much higher than a surgeon should make
